# Supplementary material for: Copper chelation redirects neutrophil function to enhance anti-GD2 antibody therapy in neuroblastoma
Source: Nat Commun. 2024 Dec 12;15:10462. doi: 10.1038/s41467-024-54689-x (PMC11638255; doi:10.1038/s41467-024-54689-x)
Supplement: Supplementary file 1 — Supplementary Information [file 41467_2024_54689_MOESM1_ESM.pdf]

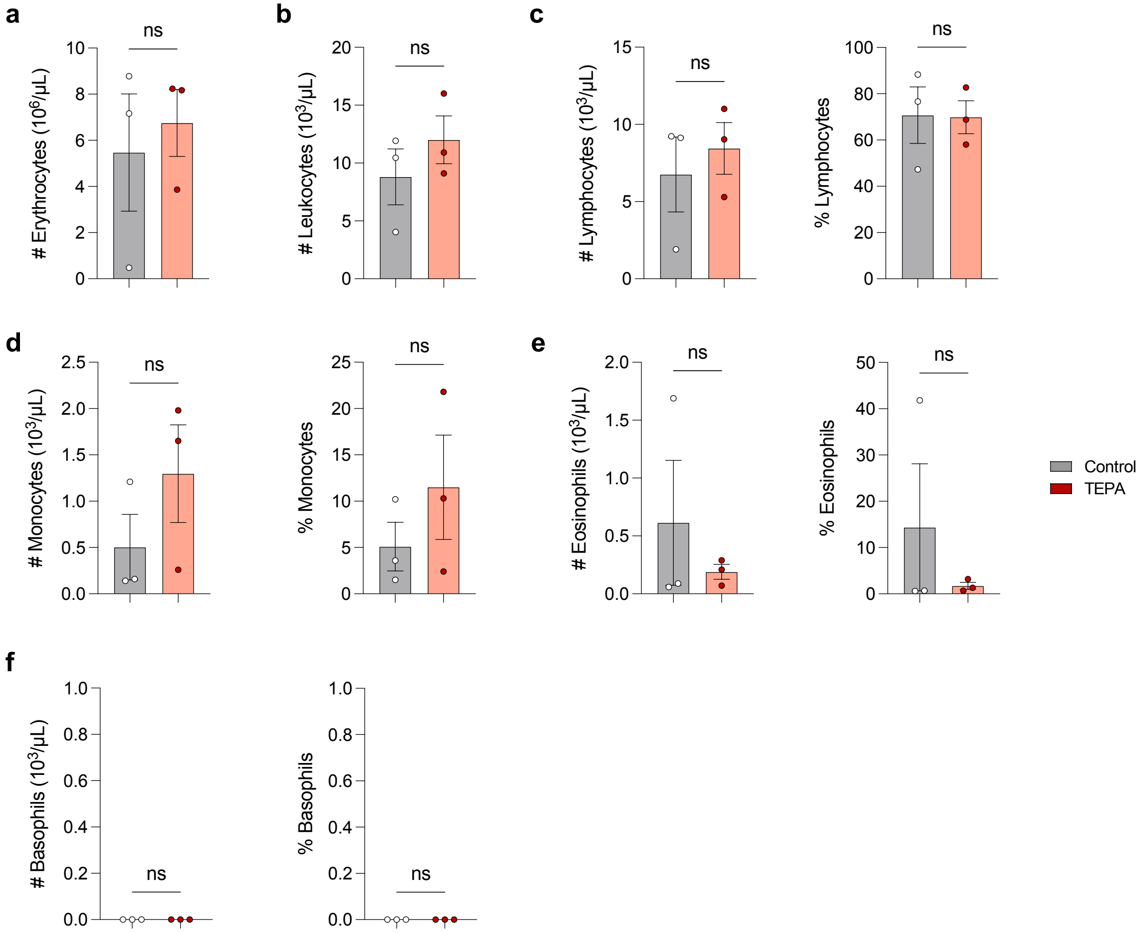

Supplementary Figure 1. Copper chelation therapy does not alter circulating numbers or frequencies of erythrocytes or non-neutrophil immune subsets. Peripheral blood obtained from *Th-MYCN* mice after one week of treatment was subjected to immunophenotyping for **a**, erythrocytes, **b**, leukocytes, **c**, lymphocytes, **d**, monocytes, **e**, eosinophils, and **f**, basophils. For **a**-**f**, data are presented as mean ± SEM, n = 3 (all groups), one independent experiment. Significance was calculated using a two-tailed Mann–Whitney U test (ns, not significant). Source data are provided as a Source Data file.

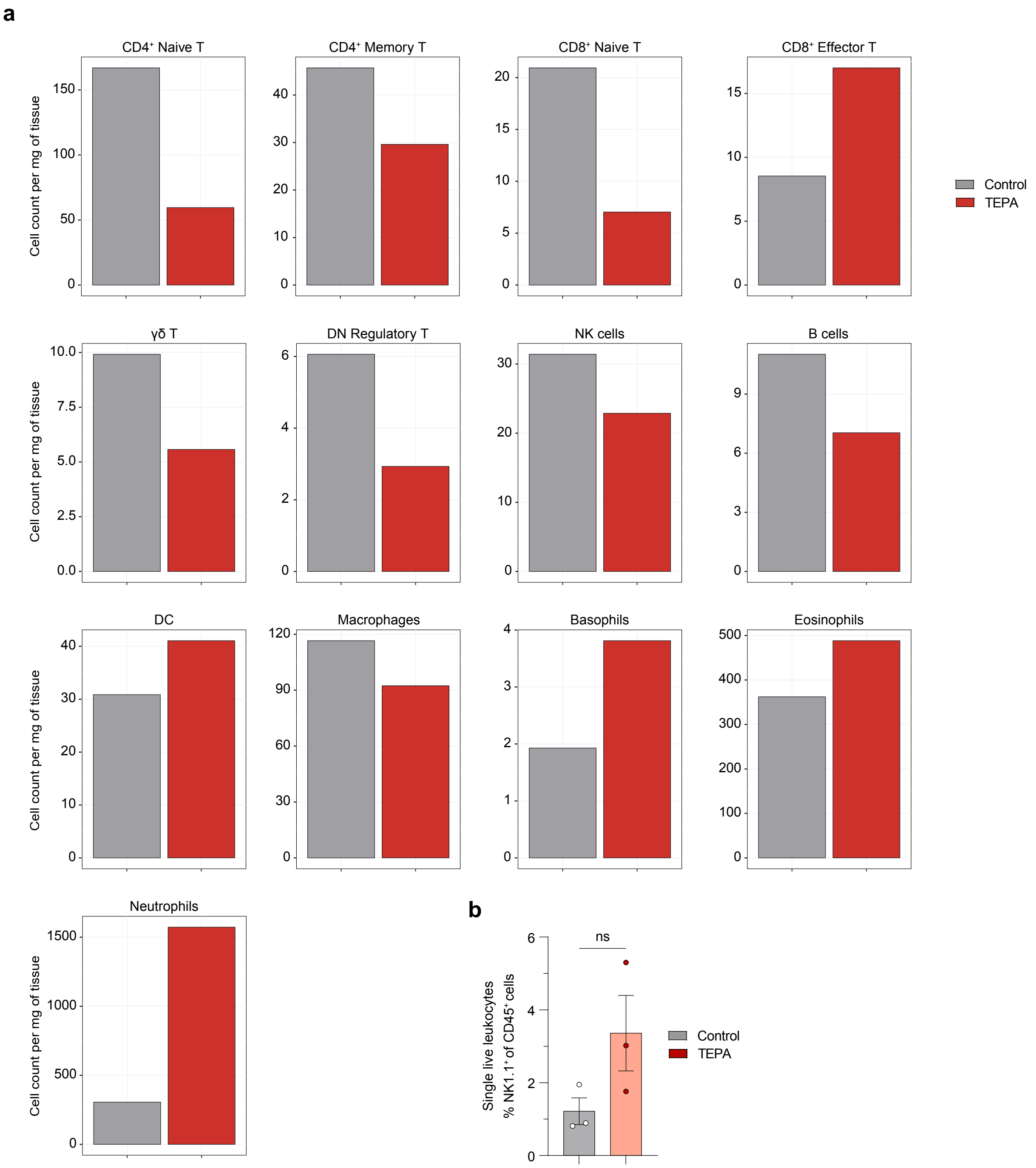

Supplementary Figure 2. Frequencies of immune subsets identified in the *Th-MYCN* neuroblastoma tumor microenvironment. **a**, Number of cells comprising immune clusters determined by single-cell RNA sequencing in control and TEPA-treated groups. Values were normalized using weight of tumor section obtained for processing. **b**, Frequency of tumor-infiltrating natural killer (NK) cells determined by flow cytometry in control and TEPA-treated groups after one week of treatment. Data are presented mean ± SEM, *n* = 3 (both groups), one independent experiment. Significance was calculated using a two-tailed Mann–Whitney *U* test (ns, not significant). Source data are provided as a Source Data file.

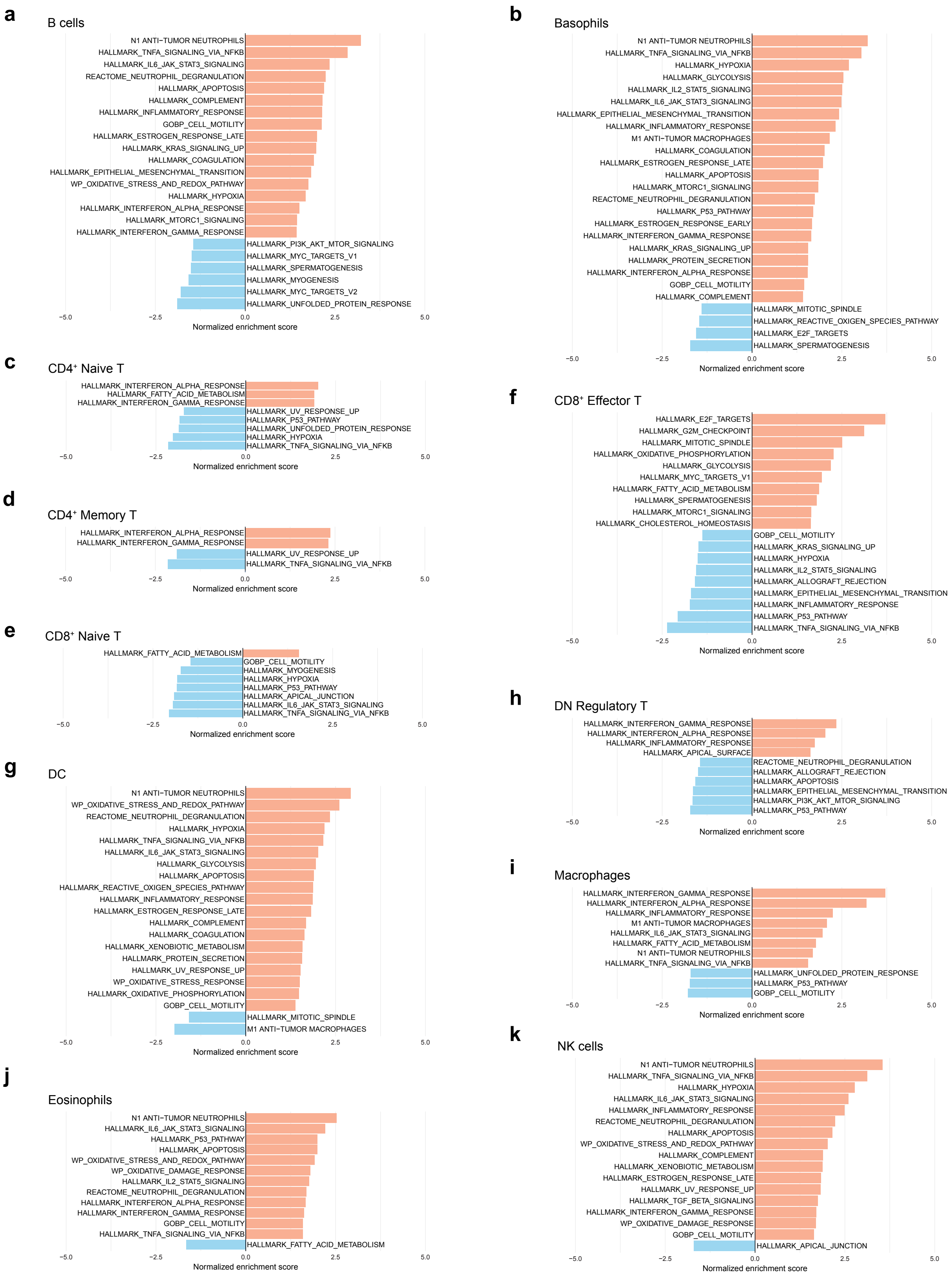

Supplementary Figure 3. Gene set enrichment analyses for immune clusters determined by single-cell RNA sequencing for **a**, B cells, **b**, basophils, **c**, CD4^{+} naive T cells, **d**, CD4^{+} memory T cells, **e**, CD8^{+} naive T cells, **f**, CD8^{+} effector T cells, **g**, dendritic cells, **h**, double-negative (DN) regulatory T cells, **i**, macrophages, **j**, eosinophils, and **k**, natural killer (NK) cells. The gamma delta (γδ) T cell cluster was excluded from analysis due to low cell numbers.

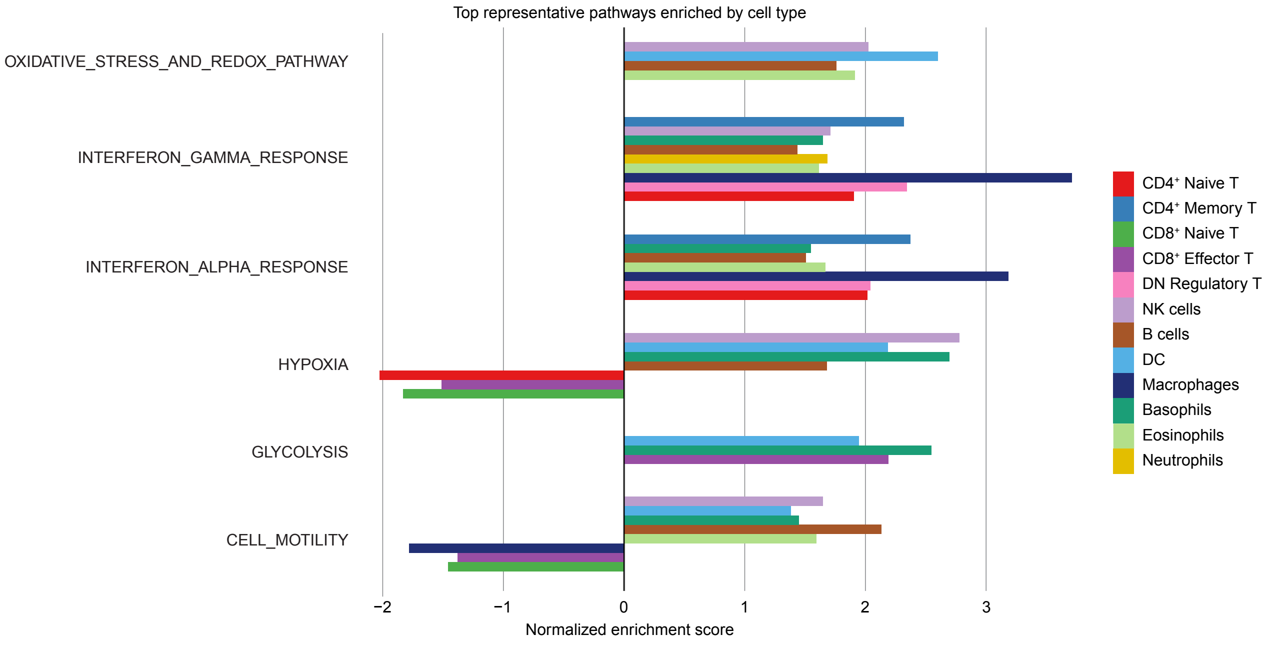

Supplementary Figure 4. Copper chelation treatment induces metabolic changes associated with a pro-inflammatory response. Bar plot displays top enriched pathways in immune clusters in TEPA-treated versus control tumors. Pathways were determined using gene set enrichment analysis as determined by single-cell RNA sequencing.

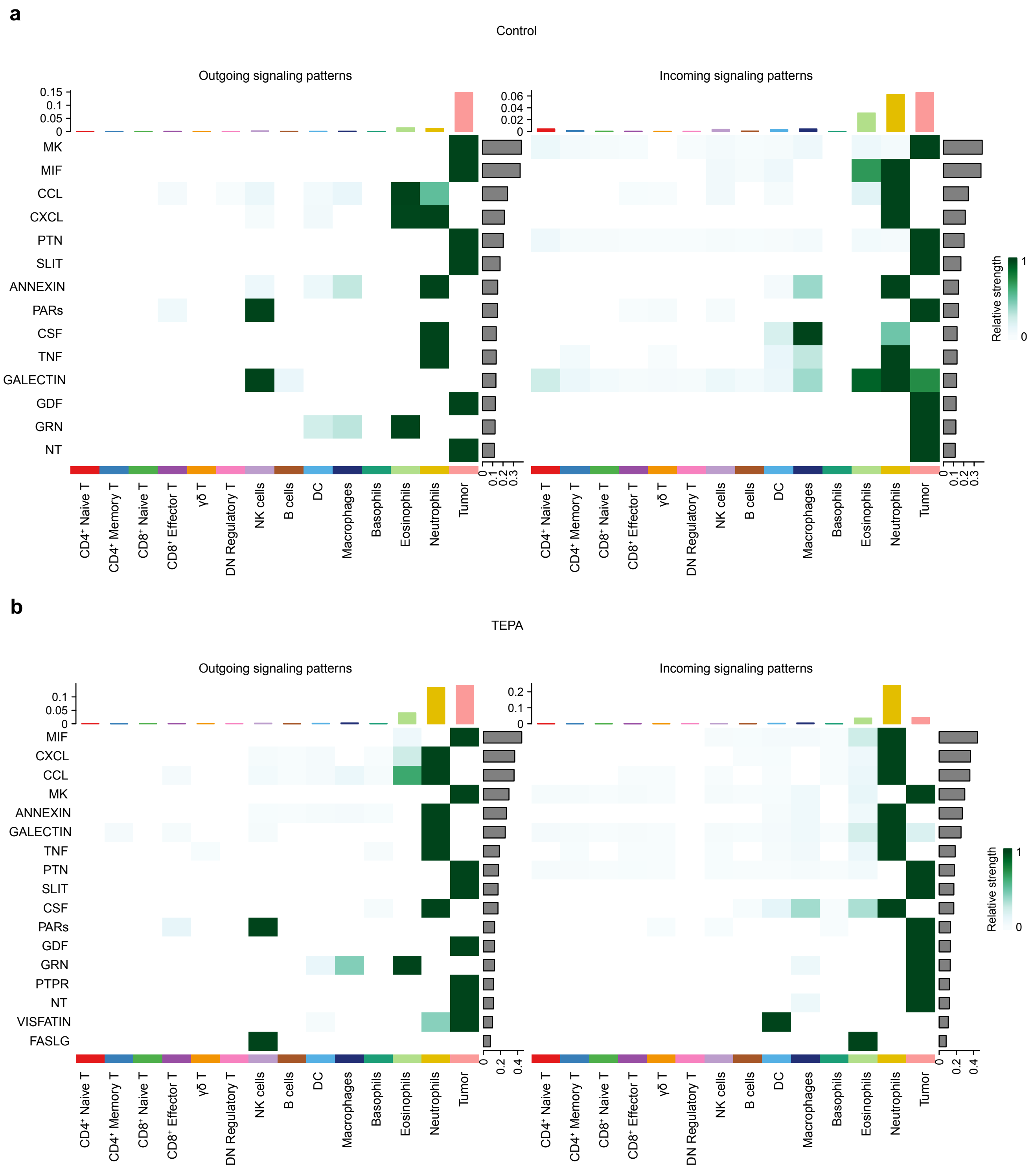

Supplementary Figure 5. Heatmaps of top signaling networks contributing to outgoing or incoming signalling of the different immune cell clusters in **a**, control and **b**, TEPA-treated tumors. Relative signal strength is represented as shades of green, the upper horizontal bar indicates the number of outgoing (left) and incoming (right) interactions per immune cell cluster, the right vertical bar indicates the relative strength of a given signaling network.

a

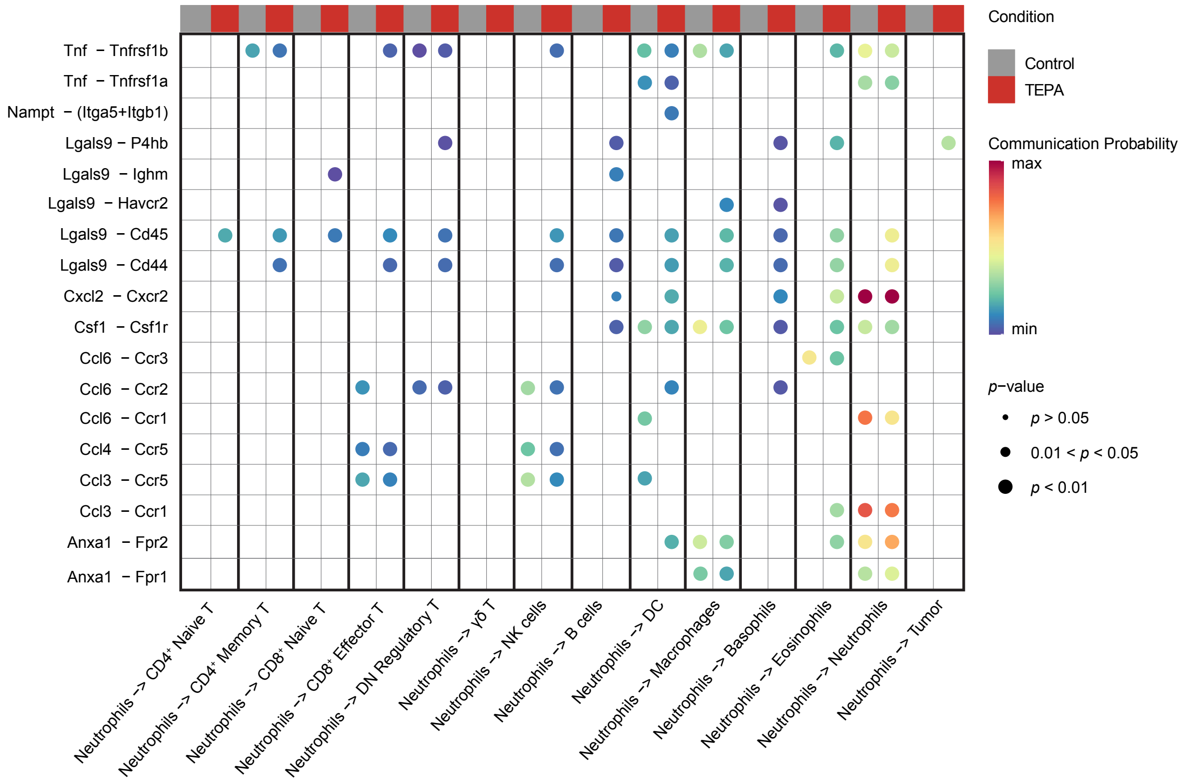

b

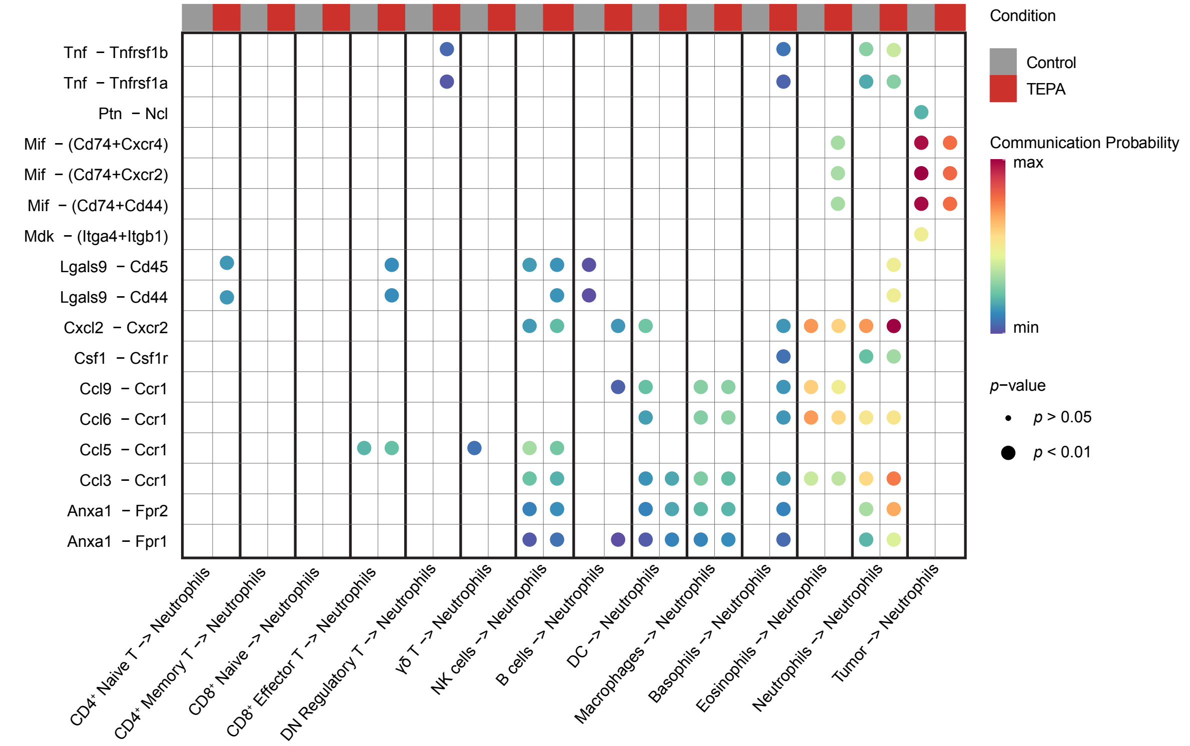

Supplementary Figure 6. Dot plots illustrating the most significant **a**, outgoing and, **b**, incoming interactions between neutrophils and the other immune cell clusters expressed as ligand-receptor pairs between control (grey) and TEPA-treated (red) groups.

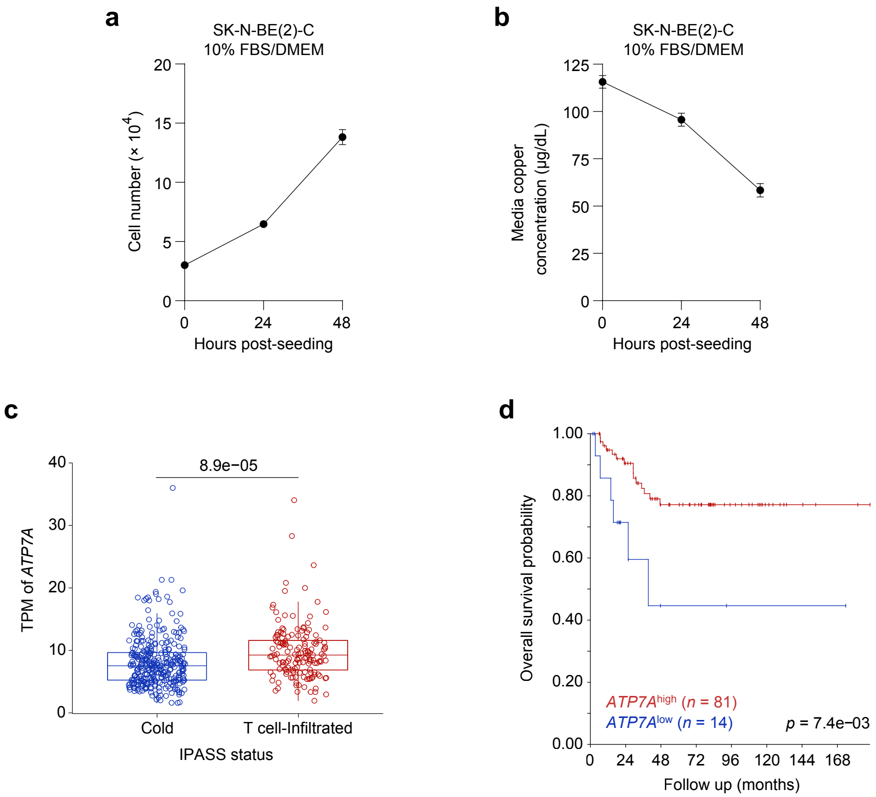

Supplementary Figure 7. Copper sequestration facilitates neuroblastoma tumor progression and is associated with decreased immune infiltration and poor prognosis. **a**, Human neuroblastoma line SK-N-BE(2)-C cell numbers were counted at timepoints as indicated with matched **b**, copper concentration in media. **c**, Box and whisker plots of transcripts per million (TPM) of copper exporter *ATP7A* and respective association with Immune Paediatric Signature Score (IPASS) status of solid human pediatric cancers (*n* = 323, cold biological replicates, *n* = 153, T cell-infiltrated biological replicates; total *n* = 476) published in[85]. Data median, and upper and lower quartiles are defined (solid lines). Significance was calculated using a two-tailed *t*-test with *p*-value displayed in figure. **d**, Kaplan–Meier analysis of overall survival in neuroblastoma cases with high (red) and low (blue) RNA levels expression of copper exporter ATP7A (total *n* = 95). Statistical comparison was calculated using a two-tailed Mantel–Cox log-rank test with *p*-value displayed in figure. Source data are provided as a Source Data file.

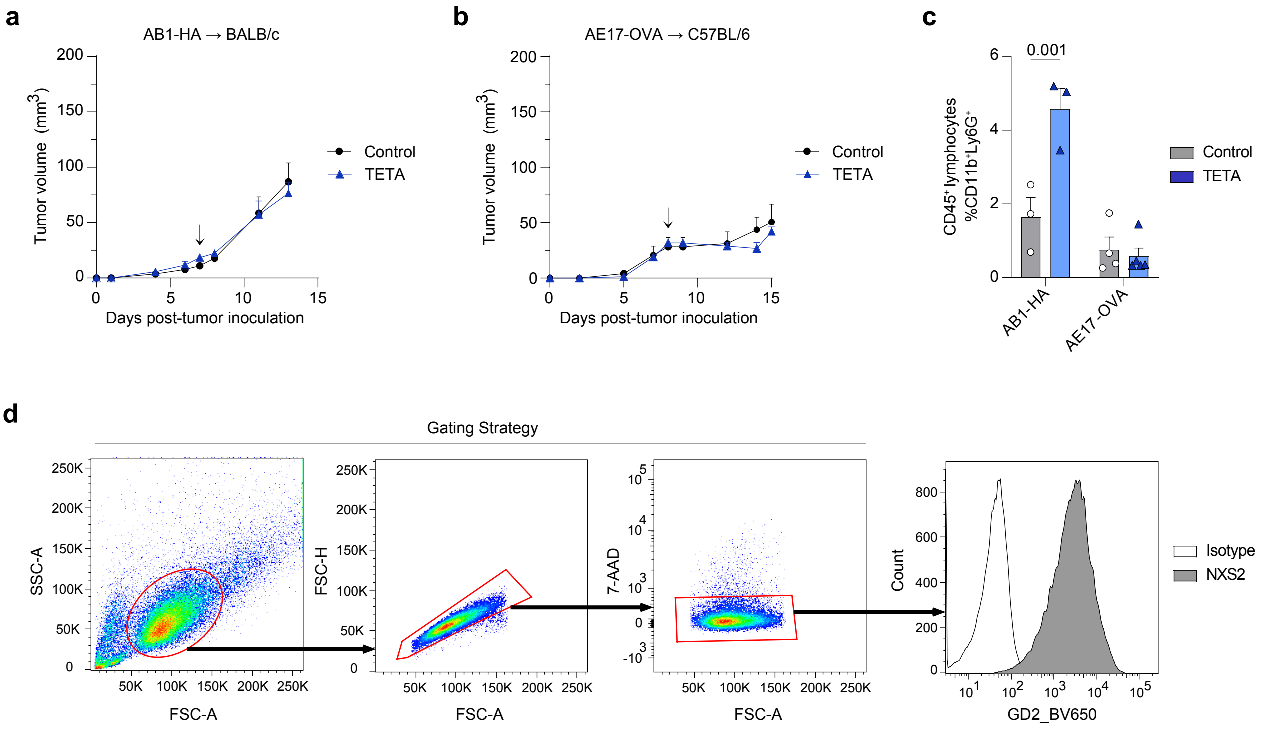

Supplementary Figure 8. Copper chelation therapy with TETA increases neutrophil infiltration in mesothelioma but only when neutrophil signaling is intact. Tumor growth kinetics in a syngeneic model of mesothelioma involving the subcutaneous inoculation of **a**, BALB/c mice with AB1-HA cells and **b**, C57BL/6J mice (which exhibit defunct neutrophil signaling demonstrated in[56]) with AE-17OVA cells. For **a**-**c**, animals commenced treatment one week after inoculation (black arrows) and were treated by oral gavage with saline (control) or TETA (400mg/kg/day) for seven days before flow cytometry analysis of tumors. **c**, Flow cytometric analysis of neutrophil frequencies in freshly dissociated tumors. Significance was calculated using a two-way ANOVA with Sidak's multiple comparisons test. For **a**-**c**, data are presented as mean + SEM, *n* = 3 (AB1-HA model), *n* = 4 (AE17-OVA model; PBS) and *n* = 5 (AE17-OVA model; TETA), one independent experiment. **d**, NXS2 cells were validated for antigenicity via flow cytometic analysis of GD2 surface expression prior to inoculation. Source data are provided as a Source Data file.

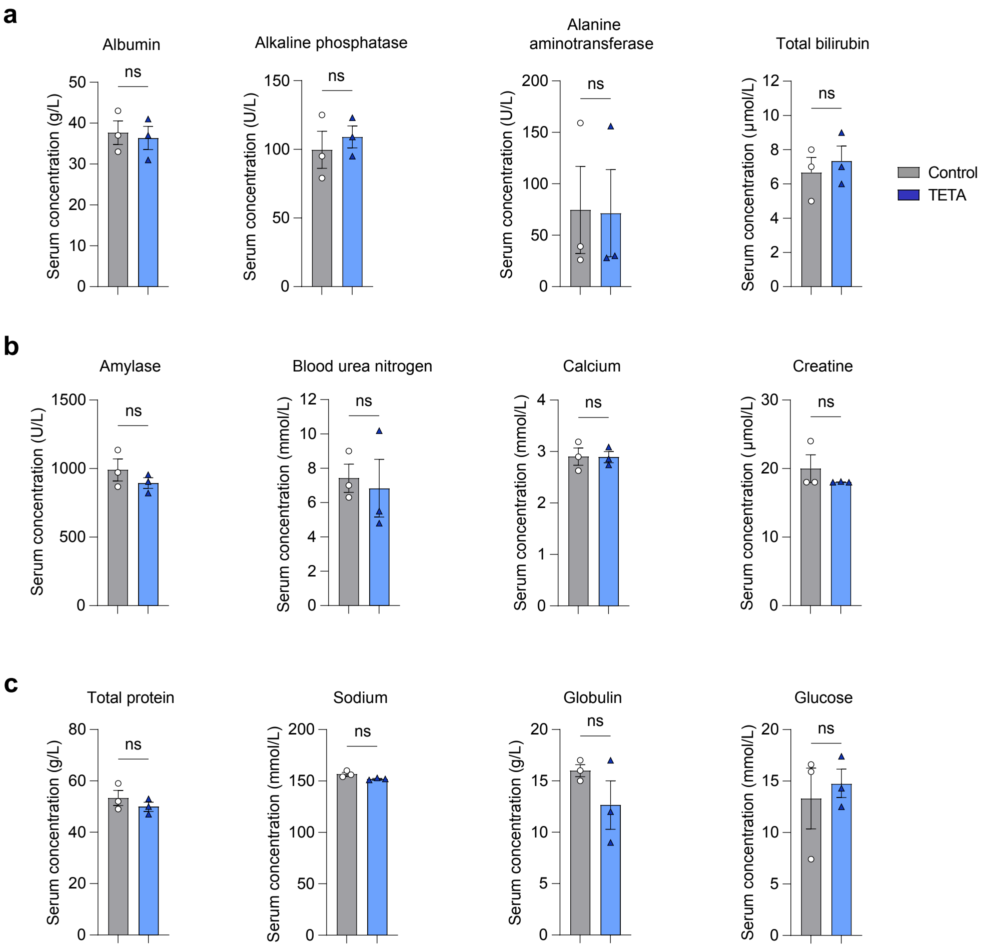

Supplementary Figure 9. Copper chelating agent TETA is a safe and non-toxic therapeutic strategy for neuroblastoma. Hematological analyses of metabolites associated with **a**, hepatic, **b**, renal, and **c**, systemic toxicities after one week of treatment. For **a**-**c**, data are presented as mean ± SEM, *n* = 3 (both groups), one independent experiment. Significance was calculated using a two-tailed Mann–Whitney *U* test (ns, not significant). Source data are provided as a Source Data file.

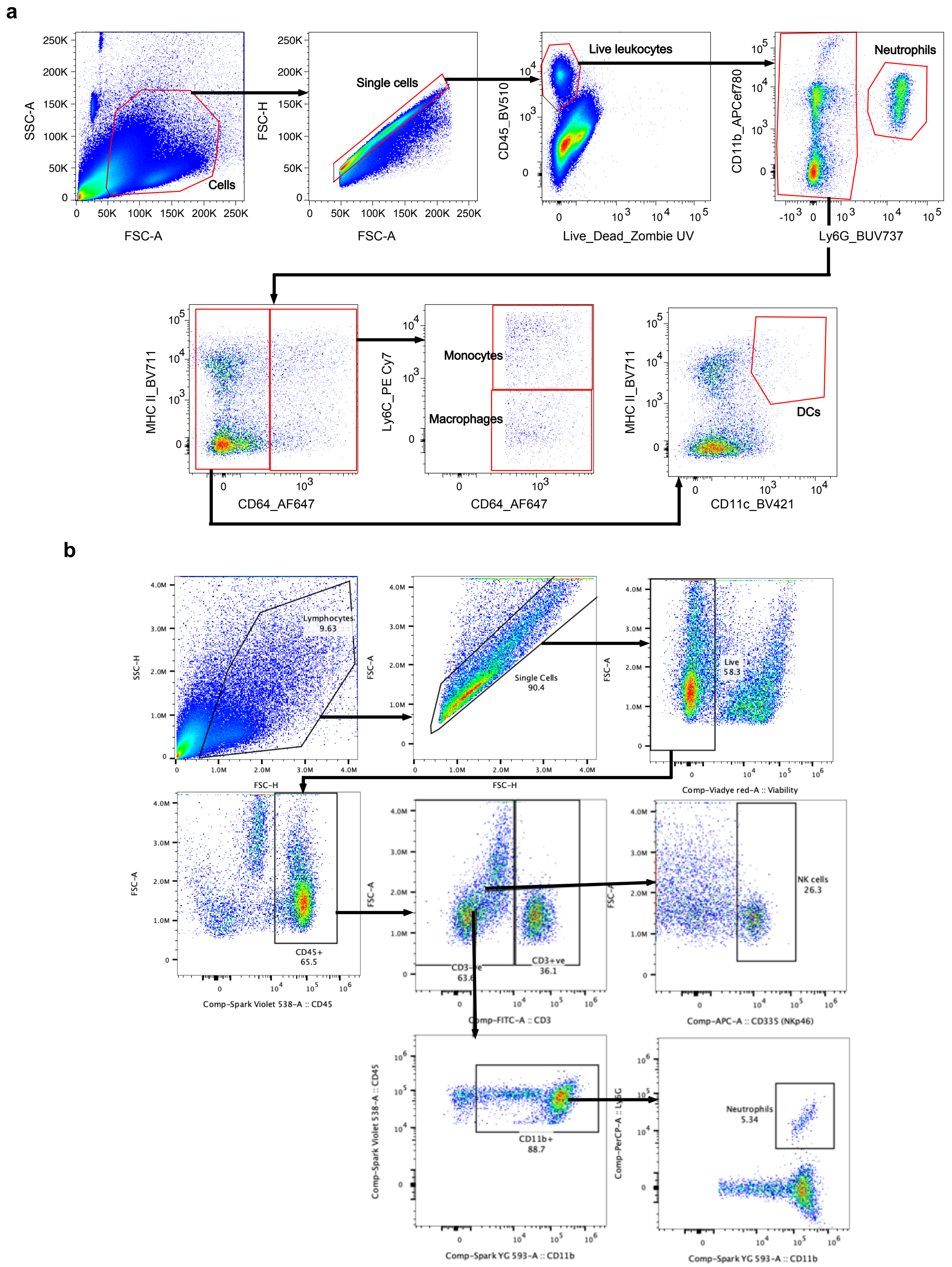

Supplementary Figure 10. Flow cytometric gating strategies used for tumoral immmunophenotyping in this study. **a**, Gating strategy used to determine frequencies of viable myeloid subsets in preclinical *Th-MYCN* tumors. **b**, Gating strategy used to determine frequencies of viable immune (lymphoid, myeloid) subsets in preclinical mesothelioma (AB1-HA; AE17-OVA) tumors.

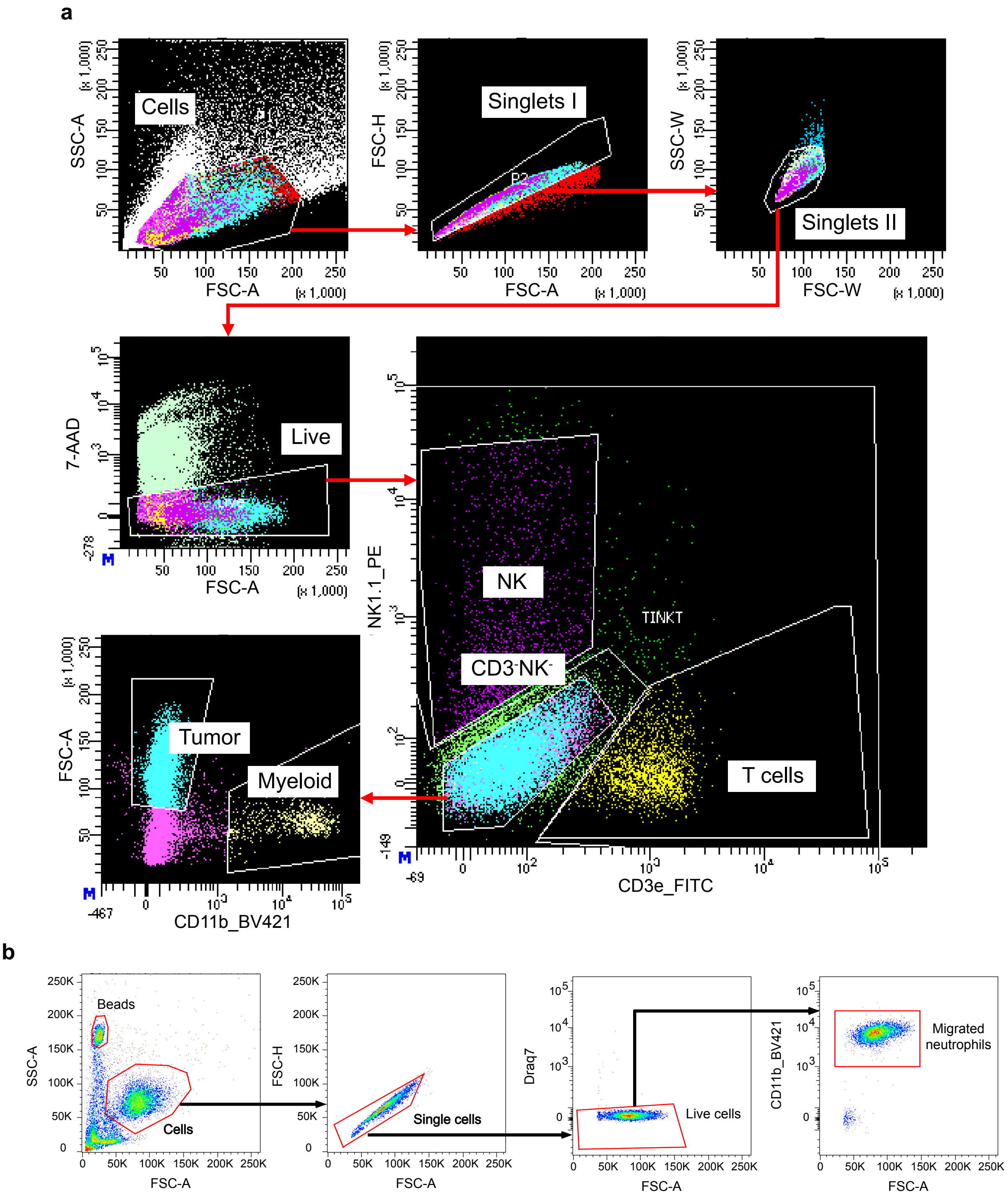

Supplementary Figure 11. Assorted flow cytometric gating strategies. **a**, Gating strategy used for single-cell RNA sequencing in *Th-MYCN* tumors to illustrate the selection of viable NK1.1$^+$ natural killer cells, CD3$^+$ T cells, CD11b$^+$ myeloid cells and the tumor compartment. **b**, Gating strategy used to migrated human neutrophils in a Transwell assay. Beads were used to count the number of neutrophils with migration calculated as a percentage of input cells (n = 400,000). Please refer to Methods for detailed description.

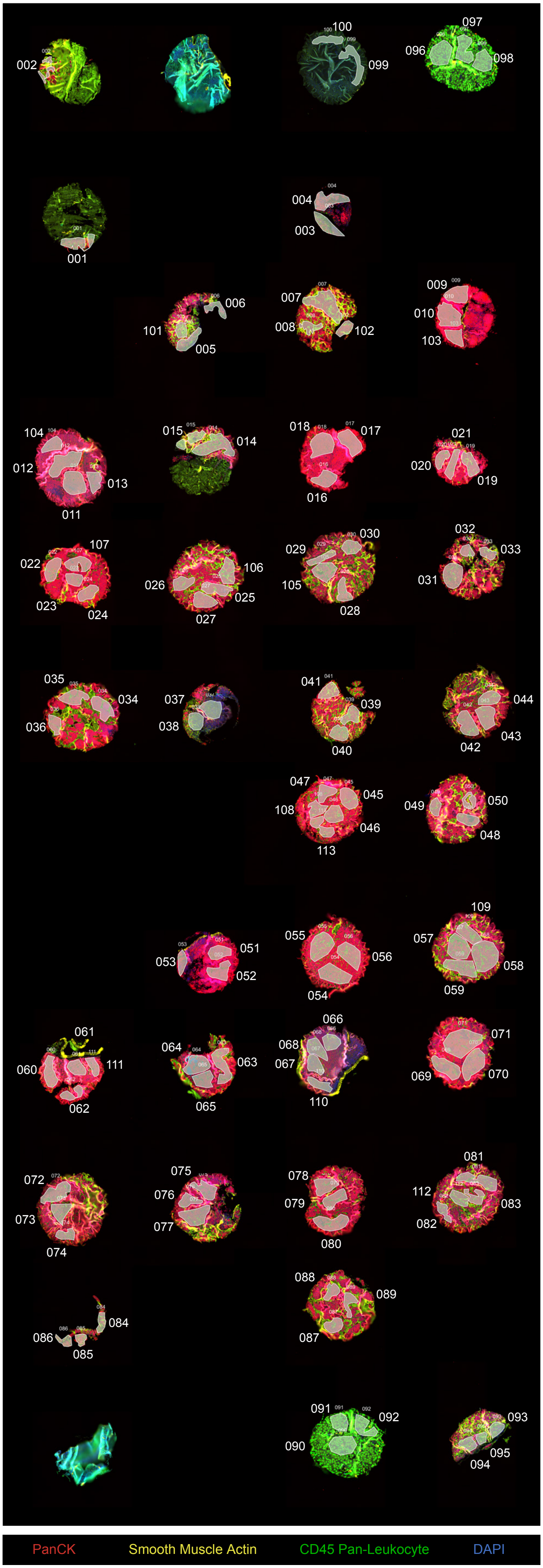

ROI_ID	Treatment	Infiltration	ROI_ID	Treatment	Infiltration
001	TEPA - 7 days	High	053	Control - 3 days	Low
002	Control - 3 days	Low	054	Control - 3 days	Low
003	TEPA - 7 days	Low	055	Control - 3 days	Low
004	TEPA - 7 days	Low	056	Control - 3 days	Low
005	TEPA - 7 days	High	057	TEPA - 7 days	Low
006	TEPA - 7 days	Low	058	TEPA - 7 days	Low
007	TEPA - 7 days	High	059	TEPA - 7 days	Low
008	TEPA - 7 days	High	060	TEPA - 3 days	High
009	Control - 3 days	Low	061	TEPA - 3 days	High
010	Control - 3 days	Low	062	TEPA - 3 days	Low
011	TEPA - 7 days	Low	063	TEPA - 3 days	High
012	TEPA - 7 days	Low	064	TEPA - 3 days	Low
013	TEPA - 7 days	Low	065	TEPA - 3 days	Low
014	TEPA - 7 days	High	066	TEPA - 7 days	High
015	TEPA - 7 days	High	067	TEPA - 7 days	High
016	TEPA - 3 days	Low	068	TEPA - 7 days	High
017	TEPA - 3 days	Low	069	TEPA - 7 days	High
018	TEPA - 3 days	Low	070	TEPA - 7 days	High
019	TEPA - 3 days	High	071	TEPA - 7 days	High
020	TEPA - 3 days	High	072	TEPA - 3 days	High
021	TEPA - 3 days	High	073	TEPA - 3 days	High
022	Control - untreated	Low	074	TEPA - 3 days	High
023	Control - untreated	Low	075	Control - 7 days	Low
024	Control - untreated	Low	076	Control - 7 days	Low
025	TEPA - 3 days	Low	077	Control - 7 days	Low
026	TEPA - 3 days	Low	078	Control - untreated	Low
027	TEPA - 3 days	Low	079	Control - untreated	Low
028	TEPA - 3 days	High	080	Control - untreated	Low
029	TEPA - 3 days	High	081	TEPA - 3 days	High
030	TEPA - 3 days	High	082	TEPA - 3 days	High
031	Control - 7 days	Low	083	TEPA - 3 days	High
032	Control - 7 days	Low	084	Control - 3 days	High
033	Control - 7 days	Low	085	Control - 3 days	High
034	Control - untreated	Low	086	Control - 3 days	High
035	Control - untreated	Low	087	Control - untreated	Low
036	Control - untreated	Low	088	Control - untreated	Low
039	Control - 3 days	Low	089	Control - untreated	Low
040	Control - 3 days	Low	101	TEPA - 7 days	High
041	Control - 3 days	High	102	TEPA - 7 days	High
042	Control - 7 days	Low	103	Control - 3 days	Low
043	Control - 7 days	Low	104	TEPA - 7 days	Low
044	Control - 7 days	Low	105	TEPA - 3 days	High
045	TEPA - 7 days	High	106	TEPA - 3 days	Low
046	TEPA - 7 days	High	107	Control - untreated	Low
047	TEPA - 7 days	High	108	TEPA - 7 days	High
048	Control - 7 days	Low	109	TEPA - 7 days	Low
049	Control - 7 days	Low	110	TEPA - 7 days	High
050	Control - 7 days	Low	111	TEPA - 3 days	Low
051	Control - 3 days	Low	112	TEPA - 3 days	High
052	Control - 3 days	Low	113	TEPA - 7 days	High

Supplementary Figure 12. Region of interest (ROI) selection and characterization of the *Th-MYCN* tissue microarray. Cores stained with fluorescently conjugated antibodies to PanCK (red), smooth muscle actin (yellow), CD45 (green) with DAPI nuclear dye. ROIs were binarily defined as having a low or high degree of CD45 immune infiltration.
