# Supplementary material for: Copper chelation redirects neutrophil function to enhance anti-GD2 antibody therapy in neuroblastoma
Source: Nat Commun. 2024 Dec 12;15:10462. doi: 10.1038/s41467-024-54689-x (PMC11638255; doi:10.1038/s41467-024-54689-x)
Supplement: Supplementary file 2 — Reporting Summary [file 41467_2024_54689_MOESM2_ESM.pdf]

nature portfolio

Corresponding author(s): Vittorio, Orazio

Last updated by author(s): Mar 1, 2024

Reporting Summary

Nature Portfolio wishes to improve the reproducibility of the work that we publish. This form provides structure for consistency and transparency in reporting. For further information on Nature Portfolio policies, see our Editorial Policies and the Editorial Policy Checklist.

Statistics

For all statistical analyses, confirm that the following items are present in the figure legend, table legend, main text, or Methods section.

n/a	Confirmed	
☐	☒	The exact sample size (n) for each experimental group/condition, given as a discrete number and unit of measurement
☐	☒	A statement on whether measurements were taken from distinct samples or whether the same sample was measured repeatedly
☐	☒	The statistical test(s) used AND whether they are one- or two-sided<br>*Only common tests should be described solely by name; describe more complex techniques in the Methods section.*
☐	☒	A description of all covariates tested
☐	☒	A description of any assumptions or corrections, such as tests of normality and adjustment for multiple comparisons
☐	☒	A full description of the statistical parameters including central tendency (e.g. means) or other basic estimates (e.g. regression coefficient) AND variation (e.g. standard deviation) or associated estimates of uncertainty (e.g. confidence intervals)
☐	☒	For null hypothesis testing, the test statistic (e.g. F, t, r) with confidence intervals, effect sizes, degrees of freedom and P value noted<br>*Give P values as exact values whenever suitable.*
☒	☐	For Bayesian analysis, information on the choice of priors and Markov chain Monte Carlo settings
☒	☐	For hierarchical and complex designs, identification of the appropriate level for tests and full reporting of outcomes
☒	☐	Estimates of effect sizes (e.g. Cohen's d, Pearson's r), indicating how they were calculated

Our web collection on statistics for biologists contains articles on many of the points above.

Software and code

Policy information about availability of computer code

Data collection	Clinical Kaplan-Meier analysis: open source, open access - available at http://r2.amc.nl.<br>The BD Rhapsody single-cell sequencing dataset was deposited on Zenodo as record 10685536.<br>The NanoString GeoMx DSP data is available on Zenodo as record 10730525.<br>The R scripts generated during this study are available at the following GitHub repository: https://github.com/antosalerno/TEPA_code_v2.0.
Data analysis	In vivo and in vitro data visualization and statistical analyses were performed using GraphPad Prism v10.

For manuscripts utilizing custom algorithms or software that are central to the research but not yet described in published literature, software must be made available to editors and reviewers. We strongly encourage code deposition in a community repository (e.g. GitHub). See the Nature Portfolio guidelines for submitting code & software for further information.

Data

Policy information about availability of data

All manuscripts must include a data availability statement. This statement should provide the following information, where applicable:

- Accession codes, unique identifiers, or web links for publicly available datasets
- A description of any restrictions on data availability
- For clinical datasets or third party data, please ensure that the statement adheres to our policy

Single-cell RNA sequencing data and NanoString GeoMx DSP data are available in Zenodo (https://zenodo.org/) using the respective reference numbers 10685536 & 10730525. The R scripts generated during this study are available in our GitHub repository at https://github.com/antosalerno/TEPA_code_v2.0.
Clinical RNA-sequencing data can be accessed via the R2 genomics analysis and visualization platform (https://r2.amc.nl). Remaining data are available within the Source Data file provided with this paper. Reagents used during the current study are available from the corresponding author on reasonable request.

Research involving human participants, their data, or biological material

Policy information about studies with human participants or human data. See also policy information about sex, gender (identity/presentation), and sexual orientation and race, ethnicity and racism.

Reporting on sex and gender	Sex was not considered in study design however all participants who volunteered peripheral blood were of the male sex.
Reporting on race, ethnicity, or other socially relevant groupings	Not relevant.
Population characteristics	Participants were healthy and had no known history of cancer or relevant disease.
Recruitment	Volunteer-based from members of immediate laboratory team.
Ethics oversight	Peripheral blood from healthy donors was obtained under ethics approved from the University of New South Wales Human Research Ethics Committee. Project Approval Numbers: iRECS0865; HC180299.

Note that full information on the approval of the study protocol must also be provided in the manuscript.

Field-specific reporting

Please select the one below that is the best fit for your research. If you are not sure, read the appropriate sections before making your selection.

[x] Life sciences [ ] Behavioural & social sciences [ ] Ecological, evolutionary & environmental sciences

For a reference copy of the document with all sections, see nature.com/documents/nr-reporting-summary-flat.pdf

Life sciences study design

All studies must disclose on these points even when the disclosure is negative.

Sample size	Experiments were performed with a minimum of two replicates biological (and a minimum of three technical replicates where relevant) as indicated in the figure legends. The sample size was determined based on standard experimental designs in this field and did not involve a pre-calculation of the sample size. A typical sample size ≥3 was chosen to ensure sufficient statistical power and to minimize random variation. Neutrophil qPCR was performed using two donors due to low RNA content obtained post-isolation.
Data exclusions	No data was excluded.
Replication	Reproducibility was ensured by at ≥3 independent replicates.
Randomization	Th-MYCN animals were randomly recruited to treatment groups as they became available for recruitment, as determined by tumor palpation. NXS2 animals were allocated to treatment groups to achieve approximately equal average initial tumor sizes and mitigate bias. Balb/c and C57BL/6 animals were randomly allocated to treatment groups.
Blinding	Blinding was not applicable in this project as our data collection and analysis were automated.

Reporting for specific materials, systems and methods

We require information from authors about some types of materials, experimental systems and methods used in many studies. Here, indicate whether each material, system or method listed is relevant to your study. If you are not sure if a list item applies to your research, read the appropriate section before selecting a response.

Materials & experimental systems

n/a	Involved in the study
☐	☒ Antibodies
☐	☒ Eukaryotic cell lines
☐	☐ Palaeontology and archaeology
☐	☒ Animals and other organisms
☐	☐ Clinical data
☐	☐ Dual use research of concern
☐	☐ Plants

Methods

n/a	Involved in the study
☐	☐ ChIP-seq
☐	☒ Flow cytometry
☐	☐ MRI-based neuroimaging

Antibodies

Antibodies used

NCR1 (EPR23097-35, Abcam, #ab233558, Lots GR3347976-1, GR3400659-6),
CD8a (EPR20305, Abcam, #ab209775, Lots GR3194554-6, GR3334378-1, GR3275780-5),
CD11b (EPR1344, Abcam, #ab133357, Lots GR3209213-12, GR3345111-10),
Mouse BD Fc CD16/CD32 Block (2.4G2, BD Pharmingen, #553142, Lot 8130843),
CD3e-FITC (145-2C11, ThermoFisher, #11-0031-82, Lot 2231864),
NK1.1-PE (PK136, ThermoFisher, #12-5941-82, Lot 2142869),
CD11b-BV421 (M1/70, BioLegend, #101251, Lot B322058),
CD45-BV510 (30-F11, BD Biosciences, #563891, Lot 1169522),
CD11b-APC-ef780 (M1/70, ThermoFisher. #47-0112-82, Lot 2272759)
Biotin-Ly6G (1A8, BioLegend, #127604, Lot B161712),
Streptavidin-BUV737 (IM7, BD Biosciences, #612775, Lot 0233661),
Pan-cytokeratin (Pan-CK) (AE1/AE3, ThermoFisher, #53-9003-82),
Smooth muscle actin (Actin) (1A4, Abcam, #ab184675),
CD45 (D3F8Q, Cell Signaling Technology, #35154),
MHC-II-BV711 (clone M5/114.15.2, BD Biosciences, #563414, Lot 1146367),
CD64-AF647 (clone X54-5/7.1, BD Biosciences, #558539, Lot 0030911),
CD11c-BV421 (clone HL3, BD Biosciences, #562782, Lot 3319725),
Ly6C-PE-Cy7 (clone AL-21, BD Biosciences, #560593, Lot 0058963).
CD45-Spark Violet (30-F11, Biolegend, #103179, Lot 7005686),
CD3-FITC (17A2, Cytek Biosciences, #35-0032-U100, Lot C0032100223353),
CD11b-Spark YG (M1/70, Biolegend, #101281, Lot 8368394),
Ly6G-PerCP (clone 1A8, Biolegend, #127653, Lot 38873),
ViaDye Red (1:1000, Cytek Biosciences, #R7-60008, Lot F-100322-02)
GD2-BV650 (clone 14.G2a, 1:150, BD Biosciences, #563705, Lot 0219086)

Validation

NCR1 was validated for immunohistochemistry with information on the manufacturer's website.
CD8a was validated for immunohistochemistry with information on the manufacturer's website.
CD11b was validated for immunohistochemistry with information on the manufacturer's website.
CD16/CD32 Block was validated by flow cytometric analysis of mouse splenocytes with information on the manufacturer's website.
CD3e-FITC was validated by flow cytometric analysis of mouse splenocytes with information on the manufacturer's website.
NK1.1-PE was validated by flow cytometric analysis of mouse splenocytes with information on the manufacturer's website.
CD11b-BV421 was validated by flow cytometric analysis of mouse bone marrow cells with information on the manufacturer's website.
CD45-BV510 was validated by flow cytometric analysis of mouse splenocytes with information on the manufacturer's website.
CD11b-APC-ef780 was validated by flow cytometric analysis of mouse bone marrow cells with information on the manufacturer's website.
Biotin-Ly6G was validated by flow cytometric analysis of mouse bone marrow cells with information on the manufacturer's website.
Streptavidin-BUV737 was validated by flow cytometric analysis of mouse splenocytes with information on the manufacturer's website.
Pan-CK was validated for immunofluorescence with information on the manufacturer's website.
Actin was validated for immunofluorescence with information on the manufacturer's website.
CD45 was validated for immunofluorescence with information on the manufacturer's website.
MHC-II-BV711 was validated by flow cytometric analysis of mouse splenocytes with information on the manufacturer's website.
CD64-AF647 was validated by flow cytometric analysis of mouse bone marrow cells with information on the manufacturer's website.
CD11c-BV421 was validated for flow cytometric analysis in the following article. PMID: 38799448.
Ly6C-PE-Cy7 was validated by flow cytometric analysis of mouse splenocytes with information on the manufacturer's website.
CD45-Spark Violet was validated by flow cytometric analysis of mouse splenocytes with information on the manufacturer's website.
CD3-FITC was validated by flow cytometric analysis of mouse splenocytes with information on the manufacturer's website.
CD11b-Spark YG was validated by flow cytometric analysis of mouse bone marrow with information on the manufacturer's website.
Ly6G-PerCP was validated by flow cytometric analysis of mouse bone marrow with information on the manufacturer's website.
ViaDye Red was validated by flow cytometric analysis of mouse splenocytes with information on the manufacturer's website.
GD2-BV650 was validated by flow cytometric analysis of human neuroblastoma cell lines information on the manufacturer's website.

Eukaryotic cell lines

Policy information about cell lines and Sex and Gender in Research

Cell line source(s)	SK-N-BE-2-C (American Type Culture Collection, #CRL-2268) is a neuroblastic cell line established from a 2yo male neuroblastoma patient.<br>Kelly is a neuroblastoma cell line established from a 1yo female neuroblastoma patient.<br>NXS2 is a murine neuroblastoma cell line derived from female A/J mice, generated and provided by Prof. Holger Lode (PMID: 9362156).<br>AB1-HA is a mouse mesothelioma cell line transfected with influenza haemagglutinin gene (HA), derived from female BALB/c mice exposed to crocidolite asbestos.<br>AE17-OVA is a mouse mesothelioma cell line transfected with full-length secretory ovalbumin (OVA) to become a neoantigen, derived from female BALB/c mice exposed to crocidolite asbestos.
Authentication	Cell lines were authenticated by STR analysis.
Mycoplasma contamination	All cell lines tested negative for Mycoplasma contamination.
Commonly misidentified lines (See ICLAC register)	No commonly misidentified cell lines were used in this study.

Palaeontology and Archaeology

Specimen provenance	*Provide provenance information for specimens and describe permits that were obtained for the work (including the name of the issuing authority, the date of issue, and any identifying information). Permits should encompass collection and, where applicable, export.*
Specimen deposition	*Indicate where the specimens have been deposited to permit free access by other researchers.*
Dating methods	*If new dates are provided, describe how they were obtained (e.g. collection, storage, sample pretreatment and measurement), where they were obtained (i.e. lab name), the calibration program and the protocol for quality assurance OR state that no new dates are provided.*

☐ Tick this box to confirm that the raw and calibrated dates are available in the paper or in Supplementary Information.

Ethics oversight	*Identify the organization(s) that approved or provided guidance on the study protocol, OR state that no ethical approval or guidance was required and explain why not.*

Note that full information on the approval of the study protocol must also be provided in the manuscript.

Animals and other research organisms

Policy information about studies involving animals; ARRIVE guidelines recommended for reporting animal research, and Sex and Gender in Research

Laboratory animals	Mus musculus; Th-MYCN (129X1/SvJ genetic background); aged 6-7 weeks were generated by Prof. William Weiss and provided by Prof. Michelle Haber.<br>Mus musculus; A/J; aged 6-7 weeks were purchased from Ozgene Animal Resources Centre, Western Australia.<br>Mus musculus; Balb/c; aged 6-7 weeks were purchased from Ozgene Animal Resources Centre, Western Australia.<br>Mus musculus; C57BL/6; aged 6-7 weeks were purchased from Ozgene Animal Resources Centre, Western Australia.
Wild animals	No wild animals were used in this study.
Reporting on sex	Concerning in vivo survival experiments, equal numbers (n=5) of male and female Th-MYCN mice were used per treatment arm (n=10 total/group) - see source data for disaggregation. Female A/J mice were used to avoid sex differences as the NXS2 cell line was originally generated using a female model (PMID: 9362156). Concerning single-cell sequencing, female Th-MYCN mice were selected to mitigate any potential differences occurring to sex. Female Balb/c and C57BL/6 mice were used due avoid sex differences as respective cell lines are both generated in female models.
Field-collected samples	No field-collected samples were used in this study
Ethics oversight	Neuroblastoma: University of New South Wales Animal Care and Ethics Committee (ACEC) (Approval numbers ACEC 20/25B, 21/96B and 18/97B). Mesothelioma: Harry Perkins Institute of Medical Research Animal Ethics Committee (Approval number AE271)

Note that full information on the approval of the study protocol must also be provided in the manuscript.

Clinical data

Policy information about clinical studies

All manuscripts should comply with the ICMJE guidelines for publication of clinical research and a completed CONSORT checklist must be included with all submissions.

Clinical trial registration	*Provide the trial registration number from ClinicalTrials.gov or an equivalent agency.*
Study protocol	*Note where the full trial protocol can be accessed OR if not available, explain why.*
Data collection	*Describe the settings and locales of data collection, noting the time periods of recruitment and data collection.*
Outcomes	*Describe how you pre-defined primary and secondary outcome measures and how you assessed these measures.*

Dual use research of concern

Policy information about dual use research of concern

Hazards

Could the accidental, deliberate or reckless misuse of agents or technologies generated in the work, or the application of information presented in the manuscript, pose a threat to:

No	Yes	
☒	☐	Public health
☒	☐	National security
☒	☐	Crops and/or livestock
☒	☐	Ecosystems
☒	☐	Any other significant area

Experiments of concern

Does the work involve any of these experiments of concern:

No	Yes	
☒	☐	Demonstrate how to render a vaccine ineffective
☒	☐	Confer resistance to therapeutically useful antibiotics or antiviral agents
☒	☐	Enhance the virulence of a pathogen or render a nonpathogen virulent
☒	☐	Increase transmissibility of a pathogen
☒	☐	Alter the host range of a pathogen
☒	☐	Enable evasion of diagnostic/detection modalities
☒	☐	Enable the weaponization of a biological agent or toxin
☒	☐	Any other potentially harmful combination of experiments and agents

Plants

Seed stocks	*Report on the source of all seed stocks or other plant material used. If applicable, state the seed stock centre and catalogue number. If plant specimens were collected from the field, describe the collection location, date and sampling procedures.*
Novel plant genotypes	*Describe the methods by which all novel plant genotypes were produced. This includes those generated by transgenic approaches, gene editing, chemical/radiation-based mutagenesis and hybridization. For transgenic lines, describe the transformation method, the number of independent lines analyzed and the generation upon which experiments were performed. For gene-edited lines, describe the editor used, the endogenous sequence targeted for editing, the targeting guide RNA sequence (if applicable) and how the editor was applied.*
Authentication	*Describe any authentication procedures for each seed stock used or novel genotype generated. Describe any experiments used to assess the effect of a mutation and, where applicable, how potential secondary effects (e.g. second site T-DNA insertions, mosiacism, off-target gene editing) were examined.*

ChIP-seq

Data deposition

☐ Confirm that both raw and final processed data have been deposited in a public database such as GEO.

☐ Confirm that you have deposited or provided access to graph files (e.g. BED files) for the called peaks.

Data access links<br>*May remain private before publication.*	*For "Initial submission" or "Revised version" documents, provide reviewer access links. For your "Final submission" document, provide a link to the deposited data.*
Files in database submission	*Provide a list of all files available in the database submission.*
Genome browser session<br>(e.g. UCSC)	*Provide a link to an anonymized genome browser session for "Initial submission" and "Revised version" documents only, to enable peer review. Write "no longer applicable" for "Final submission" documents.*

Methodology

Replicates	*Describe the experimental replicates, specifying number, type and replicate agreement.*
Sequencing depth	*Describe the sequencing depth for each experiment, providing the total number of reads, uniquely mapped reads, length of reads and whether they were paired- or single-end.*
Antibodies	*Describe the antibodies used for the ChIP-seq experiments; as applicable, provide supplier name, catalog number, clone name, and lot number.*
Peak calling parameters	*Specify the command line program and parameters used for read mapping and peak calling, including the ChIP, control and index files used.*
Data quality	*Describe the methods used to ensure data quality in full detail, including how many peaks are at FDR 5% and above 5-fold enrichment.*
Software	*Describe the software used to collect and analyze the ChIP-seq data. For custom code that has been deposited into a community repository, provide accession details.*

Flow Cytometry

Plots

Confirm that:

☒ The axis labels state the marker and fluorochrome used (e.g. CD4-FITC).

☒ The axis scales are clearly visible. Include numbers along axes only for bottom left plot of group (a 'group' is an analysis of identical markers).

☒ All plots are contour plots with outliers or pseudocolor plots.

☒ A numerical value for number of cells or percentage (with statistics) is provided.

Methodology

Sample preparation

In brief, fresh tumour sections from Th-MYCN mice were roughly minced in 6-well plates and incubated in a tumour digestion mix consisting of DMEM supplemented with 25µg/ml DNase I and 20µg/ml Collagenase IV for 1hr at 37°C at 130RPM to achieve a firm swirl using a Thermo Fisher MaxQ 6000 Shaker. A single-cell dissociation was achieved by passing the mixture through a 70µm MACS SmartStrainer (Miltenyi Biotec, Germany; #130-110-916). Cells were pelleted at 330×g for 5min and resuspended into up to 2ml of room temperature ACK Lysis Buffer (150mM NH4Cl; 1mM KHCO3; 100µM EDTA in Milli-Q water) for 3min to remove contaminating erythrocytes and neutralised with 10ml of Stain Buffer (BD Biosciences, USA; #554656). To reduce non-specific antibody staining of IgG receptors, 1 x 106 cells were pre-incubated with Mouse BD Fc CD16/CD32 Block (BD Pharmingen, USA; #553142) for 15min on ice before staining for CD3 T cells, NK1.1 NK cells and CD11B myeloid cells for 20min on ice in the dark. Tumour cells were positively selected using a previously optimised gating strategy. Approximately 50,000 single cells of each subset (CD3-NK1.1-CD11B- tumour cells, CD3+/NK1.1+ lymphocytes and CD11B+ myeloid cells) were sorted into a single tube containing foetal bovine serum (FBS; Gibco, USA, #10100-147) using the BD FACSAria III (BD Biosciences, USA).

Fresh tumor sections from NSX2-inoculated A/J mice were dissociated into single cell suspensions as previously described above for flow cytometric staining. Cells were stained in fluorescence-activated cell sorting (FACS) Buffer (1× Phosphate buffered saline [PBS]/1% FBS/0.5mM EDTA) using the following surface antibodies: CD45-BV510 (clone 30-F11, 1:250, BD Biosciences, #563891), CD11b-APC-ef780 (clone M1/70, 1:400, Thermo Fisher, #47-0112-82), Ly6G-streptavidin-BUV737 (clone 1A8, 1:300, BioLegend, #127604). After surface staining, BD Cytofix/Cytoperm Fixation/Permeabilization Kit (BD Biosciences, USA; #554714) was used according to the manufacturer's instructions, followed by intracellular staining with MMP9-APC (clone S51-82, 1:1250, StressMarq Biosciences, #SMC-396D-APC). Sample acquisition was performed using BD FACSAria III (BD Biosciences, USA) and analysed using FlowJo v10 (TreeStar, USA).

For mesothelioma tumors, fresh sections were roughly minced and incubated in a tumor digestion mix consisting of PBS/2%

	FBS supplemented with 0.1mg/mL DNase I (Worthington Biochemical, USA; #LS006331) and 1.5mg/mL Collagenase IV (Sigma-Aldrich, USA; #C4-22-1G) for 1hr at 37°C at 180RPM. Cells were strained and pelleted as above before resuspension in FACS buffer and stained for the following surface antibodies: Cells were strained and pelleted as above before resuspension in FACS buffer and stained for the following surface antibodies: CD45-Spark Violet (clone 30-F11, 1:250, Biolegend, #103179, Lot 7005686), CD3-FITC (clone 17A2, 1:500, Cytek Biosciences, #35-0032-U100, Lot C0032100223353), CD11b-Spark YG (clone M1/70, 1:400, Biolegend, #101281, Lot 8368394) and Ly6G-PerCP (clone 1A8, 1:1000, Biolegend, #127653, Lot 38873). ViaDye Red (1:1000, Cytek Biosciences, #R7-60008, Lot F-100322-02) was used as a viability marker. Samples were analysed on a Cytek 5L Aurora (Cytek Biosciences, USA) with 200,000 events collected per sample. Analyses were completed on FlowJo v10 (TreeStar, USA).<br><br>NXS2 cells were stained in FACS buffer as above and stained for surface GD2-BV650 (clone 14.G2a, 1:150, BD Biosciences, #563705, Lot 0219086) with 7-AAD used as a cell viability marker.<br><br>After incubation, all cells were collected from the bottom chamber and stained with CD11b-BV41 (clone M1/70, 1:100, BioLegend #101251) and SPHERO AccuCount Particles (Spherotech, USA, #QACBP-70-10, Lot AR01) used to determine the absolute number of migrated cells using flow cytometry.
Instrument	Flow cytometric analysis was performed using BD FACSAria III (BD Biosciences); Cytek 5L Aurora.
Software	Data was analyzed using FlowJo v10 (TreeStar, USA).
Cell population abundance	Neuroblastomas are characterized by sparse immune infiltrate and we typically obtained the following average percentages per population (per 100,000 cells acquired): 75% viable, 12% tumor, 2% natural killer, 1.5% lymphoid, 1.2% myeloid (all approximate). Mesothelioma tumors are extremely heterogeneous.
Gating strategy	Please refer to all gating strategies in Supplementary Figures 10-11.

☒ Tick this box to confirm that a figure exemplifying the gating strategy is provided in the Supplementary Information.

Magnetic resonance imaging

Experimental design

Design type	*Indicate task or resting state; event-related or block design.*
Design specifications	*Specify the number of blocks, trials or experimental units per session and/or subject, and specify the length of each trial or block (if trials are blocked) and interval between trials.*
Behavioral performance measures	*State number and/or type of variables recorded (e.g. correct button press, response time) and what statistics were used to establish that the subjects were performing the task as expected (e.g. mean, range, and/or standard deviation across subjects).*

Acquisition

Imaging type(s)	*Specify: functional, structural, diffusion, perfusion.*
Field strength	*Specify in Tesla*
Sequence & imaging parameters	*Specify the pulse sequence type (gradient echo, spin echo, etc.), imaging type (EPI, spiral, etc.), field of view, matrix size, slice thickness, orientation and TE/TR/flip angle.*
Area of acquisition	*State whether a whole brain scan was used OR define the area of acquisition, describing how the region was determined.*

Diffusion MRI ☐ Used ☐ Not used

Preprocessing

Preprocessing software	*Provide detail on software version and revision number and on specific parameters (model/functions, brain extraction, segmentation, smoothing kernel size, etc.).*
Normalization	*If data were normalized/standardized, describe the approach(es): specify linear or non-linear and define image types used for transformation OR indicate that data were not normalized and explain rationale for lack of normalization.*
Normalization template	*Describe the template used for normalization/transformation, specifying subject space or group standardized space (e.g. original Talairach, MNI305, ICBM152) OR indicate that the data were not normalized.*
Noise and artifact removal	*Describe your procedure(s) for artifact and structured noise removal, specifying motion parameters, tissue signals and physiological signals (heart rate, respiration).*

Volume censoring

Define your software and/or method and criteria for volume censoring, and state the extent of such censoring.

Statistical modeling & inference

Model type and settings

Specify type (mass univariate, multivariate, RSA, predictive, etc.) and describe essential details of the model at the first and second levels (e.g. fixed, random or mixed effects; drift or auto-correlation).

Effect(s) tested

Define precise effect in terms of the task or stimulus conditions instead of psychological concepts and indicate whether ANOVA or factorial designs were used.

Specify type of analysis: ☐ Whole brain ☐ ROI-based ☐ Both

Statistic type for inference

(See Eklund et al. 2016)

Specify voxel-wise or cluster-wise and report all relevant parameters for cluster-wise methods.

Correction

Describe the type of correction and how it is obtained for multiple comparisons (e.g. FWE, FDR, permutation or Monte Carlo).

Models & analysis

n/a	Involved in the study
☐	☐ Functional and/or effective connectivity
☐	☐ Graph analysis
☐	☐ Multivariate modeling or predictive analysis

Functional and/or effective connectivity

Report the measures of dependence used and the model details (e.g. Pearson correlation, partial correlation, mutual information).

Graph analysis

Report the dependent variable and connectivity measure, specifying weighted graph or binarized graph, subject- or group-level, and the global and/or node summaries used (e.g. clustering coefficient, efficiency, etc.).

Multivariate modeling and predictive analysis

Specify independent variables, features extraction and dimension reduction, model, training and evaluation metrics.
